# Soluble Epoxide Hydrolase Inhibition Accelerates Early Bone Repair in Critical-Size Calvarial Defects and Requires ENPP1 Activity for Osteoblast Mineralisation In Vitro

**DOI:** 10.1007/s00223-026-01635-2

**Published:** 2026-10-27

**Authors:** Bruno Sérgio Ferreira Vargas, Breno da Silva Vargas, Mariana Quirino S. Soares, Elizabeth Ferreira Martinez, Ricardo Miranda, Pedro Giorgetti Montagner, Christophe Morisseau, Francisco Humberto Nociti, Bruno Fernandes Matuck, Kevin M. Byrd, Juliana Trindade Clemente-Napimoga, Marco Antônio Húngaro Duarte, Henrique Ballassini Abdalla, Marcelo Henrique Napimoga

**Affiliations:** 1 Faculdade São Leopoldo Mandic, Instituto de Pesquisas São Leopoldo Mandic, Rua José Rocha Junqueira, 13 – Swift, Campinas, SP 13405-755 Brazil; 2 Department of Entomology and UCD Comprehensive Cancer Center, University of California, Davis, CA USA; 3 University of Maryland, Baltimore, MD USA; 4 Philips Institute for Oral Health Research, Virginia Commonwealth University, Richmond, VA USA; 5 Department of Restorative Dentistry, Endodontics and Dental Materials, Bauru School of Dentistry, Universidade de São Paulo (USP), Bauru, SP Brazil

**Keywords:** Bone regeneration, Soluble epoxide hydrolase, ENPP1, Biomineralization, Critical-size defect, Spatial transcriptomics, TPPU

## Abstract

Critical-size craniofacial bone defects heal poorly, and pharmacological strategies capable of accelerating bone repair remain limited. Soluble epoxide hydrolase (sEH) degrades epoxyeicosatrienoic acids (EETs), lipid mediators associated with inflammation resolution; however, the osteoblastic effectors that translate sEH inhibition into matrix mineralisation are unknown. We investigated whether pharmacological sEH inhibition accelerates craniofacial bone repair, and whether ectonucleotide pyrophosphatase/phosphodiesterase 1 (ENPP1) a candidate regulator of pyrophosphate metabolism participates in this response. In vivo, bilateral 5-mm critical-size calvarial defects were created in male Wistar rats (n = 6 per group) treated with the sEH inhibitor TPPU (10 mg/kg/day, p.o.) or vehicle for 14 or 28 days. Repair was assessed by micro-computed tomography, histomorphometry, sEH immunohistochemistry and multiplex spatial RNA in situ hybridisation (RNAscope) of a predefined six-gene osteogenic panel (*Spp1, Runx2, Col1a1, Alpl, Bglap, Enpp1*). In vitro, human SAOS-2 osteoblastic cells were treated with TPPU and/or the ENPP1 inhibitor STF-1084 to assess viability, osteogenic gene expression, ENPP1 expression and enzymatic activity, alkaline phosphatase (ALP) activity and mineralised matrix formation. At 14 days, TPPU increased BV/TV, BS/TV and trabecular number and reduced trabecular separation and total porosity (*p* < 0.05); these differences were no longer detected at 28 days, whereas histomorphometry showed a greater newly formed bone area in TPPU-treated animals at both time points. sEH immunolabelling was reduced by TPPU at 14 and 28 days (*p* < 0.001), confirming target engagement. Spatially, Enpp1-positive cells were more abundant in TPPU-treated tissue at 14 days, the only panel gene reaching significance at the per-cell level. In vitro, TPPU increased ENPP1 mRNA and protein. STF-1084 dose-dependently reduced ENPP1 activity, ALP activity and mineralised matrix deposition without affecting viability, and co-treatment with TPPU did not restore mineralisation despite preserved or increased OSX, RUNX2 and BSP expression. Pharmacological sEH inhibition accelerates the early phase of craniofacial bone repair and is associated with increased ENPP1 expression in regenerating tissue. In human osteoblastic-SAOS cells, ENPP1 enzymatic activity is necessary for mineralisation and for the pro-mineralising effect of sEH inhibition, identifying ENPP1 as a candidate downstream effector of the sEH/EpFA axis and a target for further genetic validation.

## Introduction

Critical-size bone defects in the craniomaxillofacial region remain a clinical and economic burden, requiring three-dimensional structural restoration that the natural healing response cannot accomplish unaided [1]. Although autografts, allografts, xenografts, alloplastic substitutes and guided bone regeneration succeed in small defects, large skeletal voids continue to fail or relapse with high reoperation rates [2–4]. The induced membrane technique remains imperfect, and even refined regenerative strategies still face moderate success rates and considerable risk of failure [5, 6].

Bone healing begins with an inflammatory phase in which IL-1β, TNF-α and matrix metalloproteinases recruit inflammatory and progenitor cells and remodel the provisional matrix. These same mediators, however, suppress osteoblast function and matrix deposition when their activity is prolonged, and their timely restraint by endogenous inhibitors such as IL-1RA, IL-10 and TIMPs is required for the transition to ossification [5]. This dual role explains why strategies that simply neutralise individual cytokines have yielded inconsistent results in fracture and critical-size defect models: the target is not inflammation itself, but the timing of its resolution. Resolution is an actively regulated, lipid-mediated process. Polyunsaturated fatty acid–derived specialized pro-resolving mediators and epoxy-fatty acids terminate inflammatory signalling and promote tissue repair, and their inadequate production compromises bone regeneration [7–9]. Excess pro-inflammatory eicosanoids, derived from oxidative metabolism of arachidonic acid, further suppress osteoblast function and matrix deposition [10].

Among the enzymes that control this network, soluble epoxide hydrolase (sEH) is particularly attractive pharmacologically, it degrades epoxyeicosatrienoic acids (EETs) into less bioactive diols, so that its inhibition raises the local tone of endogenous pro-resolving and pro-angiogenic mediators rather than introducing an exogenous agonist. In a critical-size defect, where the inflammatory phase is prolonged and spontaneous healing fails, this upstream, resolution-promoting mechanism is a rational and clinically tractable intervention [11–13]. Pharmacological inhibition of sEH protects alveolar bone in periodontitis, preserves joint integrity in collagen-induced arthritis, and shifts the immune balance toward Treg/M2-like phenotypes with enhanced production of MaR1 and LXA4 [7, 14–18]. Clinically, elevated sEH expression and activity correlate with stage III/IV grade C periodontitis severity [18], and orally active sEH inhibitors (TPPU and EC5026) have advanced toward analgesic and anti-inflammatory clinical use [19–21].

Whereas sEH inhibition has been shown to prevent inflammatory bone loss, its capacity to drive bone formation in large skeletal defects, and the molecular effectors that translate sEH inhibition into mineralization, remain poorly defined. A recent in vitro study indicates that sEH inhibition stabilizes osteogenic EETs and supports osteoblast activity [22], and that sEH inhibition also restrains osteoclast differentiation [23] and rescues bone in periodontitis-associated estrogen deficiency [24]. EETs further engage the ROBO1/HIF-1α axis to couple osteogenesis with angiogenesis [25, 26] and support the vascular component of bone repair via EMCN regulation [27]. However, it remains unclear whether sEH inhibition promotes matrix mineralization through a specific downstream osteoblastic effector.

Ectonucleotide pyrophosphatase/phosphodiesterase−1 (ENPP1) is one of the principal regulators of pyrophosphate metabolism at the osteogenic surface [28]. Beyond its pyrophosphate-generating activity in cementum and vasculature, ENPP1 is required for osteoblast differentiation, with loss of ENPP1 reducing osteoblastic gene expression and matrix maturation [29]. This dual, context-dependent biology—mineralization broke at the matrix level, but maturation factor at the osteoblast level—has not previously been connected to the sEH/EpFA axis.

Thus, we hypothesised that pharmacological sEH inhibition would enhance bone neoformation in critical-size calvarial defects and would engage an ENPP1-associated osteogenic program in osteoblastic cells. Inflammatory endpoints were not assessed in the present study.

## Materials and Methods

### Chemicals and Reagents

TPPU[1-(1-propanoylpiperidin-4-yl)-3-[4-(trifluoromethoxy)phenyl]urea] was synthesized at the Department of Entomology and Nematology, University of California-Davis, USA, as previously described [30]. For in vivo administration, TPPU was dissolved in polyethylene glycol 400 (PEG400; Sigma-Aldrich, St. Louis, MO, USA) at 10 mg/kg by sonication. The TPPU dose was selected based on previous dose–response studies in which 10 mg/kg/day demonstrated optimal protective effects against alveolar bone loss [7, 31].

For in vitro experiments, TPPU was dissolved in dimethyl sulfoxide (DMSO) as a stock solution and subsequently diluted in cell culture medium to the appropriate working concentrations. Furthermore, the selective ENPP1 inhibitor, STF-1084 (SML3553, lot# 0000409735, Sigma-Aldrich, St. Louis, MO, USA), was used in vitro at 0.5–100 μM and was dissolved in appropriate cell culture medium.

### Animal Model and Experimental Design

Adult male Wistar rats (7 weeks; 200–240 g; ANILAB, Paulínia, SP, Brazil) were housed four per cage at 23 ± 1 °C on a 12:12 h light/dark cycle (lights on 06:00) with ad libitum access to food and water and acclimated for ≥ 1 week. All procedures were approved by the Institutional Animal Research Ethics Committee (CEUA/SLMANDIC #2022/025) and conducted in accordance with CONCEA guidelines and the ARRIVE 2.0 reporting. The sample size estimation (n = 6 per group) was derived from a 95% confidence interval, a maximum acceptable deviation of 0.22, a minimum standard error of 5% and α = 0.05 [32]. Animals were randomly assigned (Randomization.com) to TPPU (10 mg/kg/day, p.o.) or vehicle (PEG400/saline) for 14 or 28 days. Animals were euthanized under deep anesthesia, with cervical dislocation performed as a secondary method to ensure death at the respective experimental time points. Additionally, all surgical procedures, treatment administration, outcome assessments, and experimental readouts were performed and analyzed under blinded conditions with respect to group allocation.

### Surgical Procedures

Anesthesia was induced with 5% ketamine hydrochloride and 2% xylazine hydrochloride. After dorsal cranial trichotomy and antisepsis with 1% povidone-iodine, a 2 cm midline longitudinal incision exposed the parietal bone. A bilateral 5-mm bicortical critical-size defect was created with a trephine drill (ref. 103.207, Neodent, Curitiba, Brazil) under continuous sterile saline irrigation; the dura mater was preserved. Defects were covered with a resorbable collagen membrane (Geistlich Brasil, SP, Brazil) to standardize healing. Wounds were closed with 4–0 polyglactin 910 (Vicryl®, Ethicon). Postoperative care included enrofloxacin (2.5%, 2 mg/kg) and tramadol (5 mg/kg, every 12 h for 3 days).

### Micro-Computed Tomography (Micro-CT)

Samples were scanned using a SkyScan 1174 micro-computed tomography system (Bruker, Belgium) at 50 kV and 800 µA, with a 306° rotation and an angular step of 0.5°. The scans were acquired with an isotropic voxel size of 19.7 µm. A 0.5 mm aluminum filter was applied to reduce artifacts. All images were reconstructed using NRecon software (Bruker, Belgium). For analysis, defects were manually registered using DataViewer software (Bruker, Belgium). A standardized region of interest (ROI) was defined, covering the entire 5-mm diameter of the defect and the full thickness of the calvarial bone at the defect site. This ROI was individually adapted for each sample. Image segmentation was performed by a single evaluator using a semi-automated threshold algorithm in CTAnalyser software (Bruker, Belgium). Morphometric parameters were then calculated, including bone volume fraction (BV/TV, %), total porosity (Po[tot], %), bone surface density (BS/TV, %), trabecular thickness (Tb.Th, mm), trabecular number (Tb.N, 1/mm), trabecular separation (Tb.Sp, mm), and connectivity density (Conn.Dn, 1/mm^3^). All measurements were calculated according to ASBMR guidelines [33].

### Histology and Histomorphometry

Specimens were demineralized in 20% formic acid (≈5 days), dehydrated and paraffin-embedded. Serial 4-µm sections taken from the central region of the defect were stained with hematoxylin and eosin and imaged with an Axioskop 2 Plus / AxioVision Rel. 4.8 system (Carl Zeiss, Germany). Bone neoformation area was quantified using ImageJ 152r by two independent, calibrated, blinded examiners (R.M., E.F.M.).

### Immunohistochemistry

Tissues were snap-frozen in liquid nitrogen, fixed in 10% formaldehyde for 72 h, embedded in paraffin and sectioned at 4 µm. Endogenous peroxidase was quenched (3% H_2_O_2_, three times, 5 min each). After 5% skim-milk blockade and citrate-buffer antigen retrieval (pH 6.0, 97 °C, 20 min), sections were incubated overnight at 4 °C with anti-sEH (1:100; gift from Dr. B.D. Hammock). Detection used the Advance HRP system (Dako) with DAB chromogen and Mayer’s hematoxylin counterstain. Negative controls omitted the primary antibody; rat liver served as positive control [12]. Quantitative scoring was performed at 100× magnification by a blinded observer.

### *Multiplex RNA *In Situ* Hybridization (RNAscope) and Computational Analysis*

All reagents mentioned in this protocol were obtained from ACD and used without additional processing unless specified otherwise. The six-gene panel was defined a priori to cover osteoprogenitor commitment (*Runx2*), matrix production and maturation (*Col1a1, Spp1, Bglap*), matrix-vesicle-associated mineralisation (Alpl) and extracellular pyrophosphate metabolism (*Enpp1*), the latter selected because ENPP1 is a principal regulator of pyrophosphate at the osteogenic surface and is required for osteoblast differentiation, and because its relationship with the sEH/EpFA axis had not previously been examined.

The sample underwent standard deparaffinization and rehydration procedures. A single drop of RNAscope hydrogen peroxide was applied to each slide and incubated at room temperature (RT) for 10 min. The excess reagent was removed by gently tapping off the slides. Samples then underwent antigen retrieval followed by drying, as per standard protocol. Concurrently, the HybEZ oven was preheated and set to the RNAscope setting (40 °C). Humidity chambers were prepared by moistening hydration paper with nuclease-free water. A hydrophobic barrier was drawn around each tissue section using an Immedge pen and allowed to dry at RT for 5 min. Slides were then mounted into the slide holder.

Each tissue section was treated with one drop of Protease IV, and the holder was placed back into the tray and incubated in the HybEZ oven for 30 min. Meanwhile, a 1X RNAscope wash buffer solution was prepared using nuclease-free water. Probe hybridization mixes were prepared by combining 1 µL of T probe with 100 µL of probe diluent. After incubation, the slide tray was removed, and the slide holder was washed in buffer twice for 2 min each. The slides were dried, and a paper towel was used to absorb any excess liquid surrounding the hydrophobic barrier.

Next, 20 µL of the hybridization solution, or a drop of control probe (positive or negative), was added to each tissue area. Slides were returned to the tray and incubated in the HybEZ oven for 2 h. Post-hybridization, slides were washed again (2 × 2 min) in wash buffer. Signal amplification steps were performed sequentially using AMP1, AMP2, and AMP3 reagents, each for 30 min, followed by washing between each step. Fluorophores T1 through T4 were then applied, incubated for 15 min, and washed.

Subsequently, RNAscope DAPI stain was applied to each section for 30 s. Excess DAPI was removed by tapping, and slides were immediately mounted using Prolong Gold Antifade. Imaging was carried out using a Leica DMi8 equipped with a THUNDER Imager (Leica Microsystems), utilizing either a 40× or 63× 1.35 NA oil immersion objective.

After the imaging of T1–T4 probes was completed, the next probe sets (T5–T8 and T9–T12) required removal of the prior fluorescent signals. Slides were placed in 4X saturated sodium citrate (SSC) solution overnight or until the cover glass could be detached without force. During this interval, a 10% dilution of RNAscope cleaving solution was prepared using 4X SSC. Once the coverslips were removed, slides were placed in a slide holder, and one drop of the cleaving solution was applied to each sample region. The holder was inserted into the tray and incubated in the HybEZ oven for 15 min. Afterwards, the slides were washed in 0.5% Tween solution, two times for 2 min each. This cycle was repeated before proceeding.

The T5–T8 probes were then applied to the samples as previously described, and imaging was performed. Following imaging, the reporter signals from T5–T8 were removed using the same cleaving protocol, hydrogen peroxide treatment was omitted, and Protease III was used in place of Protease IV. An additional step was required between AMP3 and fluorophore application: the use of FFPE reagent. A 1:40 dilution was prepared by mixing 2.5 µL of FFPE reagent with 100 µL of 4X SSC. After washing slides with 1X wash buffer, the diluted FFPE reagent was added to each sample region and incubated at room temperature for 30 min. Following incubation, slides were rinsed in 1X wash buffer before proceeding with fluorophore addition. A multi-phase workflow was used to process RNA multiplex imaging data following acquisition with Fiji (ImageJ). The raw.lif files were first converted into 8-bit images and saved in the ome.tiff format. Imaging was conducted in three sequential rounds, with four probe sets used per round on the same section, resulting in a 9-channel dataset. These image sets were aligned using the Warpy and image combiner tools to generate a unified 9-plex image stack. For segmentation, two pretrained deep learning models were applied: Cellpose 2.0 for the 9-plex ISH dataset and StarDist for other datasets. The segmentation approach was iteratively optimized using a set of 80 fluorescent tissue images from archived human samples. Training iterations continued until reliable delineation of individual cell boundaries was achieved in the relevant tissue layers. The transcript quantification was performed as described [34].

### Cell Culture, Drug Supplementation and Cell Viability

Human osteoblastic SAOS-2 cells (ATCC) were maintained in McCoy’s 5A (Sigma) supplemented with 15% FBS, 100 IU/mL penicillin, and 50 µg/mL streptomycin (Sigma) at 37 °C, 5% CO_2_. For osteogenic differentiation, cells were switched to medium with 10% FBS, 10 mM β-glycerophosphate (Sigma) and 50 µg/mL ascorbic acid (Gibco). TPPU ranging from 1 to 1000 µM (kindly provided by Hammock’s Lab), and STF-1084 ranging from 0.5 to 100 µM (SML3553, Sigma-Aldrich) were freshly diluted in culture medium. Mitochondrial activity / cell viability was assessed by MTT assay at 1, 3, 5, 7 and 10 days.

### Alkaline Phosphatase Activity

Alkaline phosphatase (ALP) activity was estimated by the release of thymolphthalein from thymolphthalein monophosphate using a commercial kit (Labtest Diagnostica SA, Lagoa Santa, MG, Brazil). Briefly, the culture medium was removed, and the wells were washed three times with PBS (Gibco) at 37 °C. Subsequently, they were filled with 2 ml of 0.1% sodium lauryl sulfate (Sigma) and left for 30 min at room temperature. Then, 50 μL of thymolphthalein monophosphate was blended with 0.5 mL of diethanolamine buffer, 0.3 mmol/mL, pH 10.1, and left for 2 min at 37 °C. After this period, 50 μL of the sample was taken from each well. This stood for 10 min at 37 °C, and then 2 mL of a solution of Na_2_CO_3_ 0.09 mmol/mL and NaOH 0.25 mmol/mL was added to allow color development. After 30 min, absorbance was measured at 590 nm, and ALP activity was calculated from a standard curve. Results were calculated, and data were expressed as ALP activity normalized by the total protein content (Pierce BCA Protein Assay Kit).

### Alizarin Red Stain

The Alizarin Red stain (Sigma) assessed the mineralized matrix formation. The mineralization process was quantified on days 14 and 21. For that, the cells were initially fixed in 1.33 M formalin at room temperature for two hours. Then, the cells were dehydrated through a graded series of alcohol and stained with 0.02 g/ml of Alizarin Red S (pH 4.2) for 10 min. Subsequently, the cells were thoroughly washed with deionized water to remove excess dye, and the microplates were left to dry. Once parched, 280 μL of 10% acetic acid was added to each well and placed in the microplate shaker for 30 min at room temperature. Thereupon, the contents of each microplate were transferred to polypropylene tubes and heated at 85 °C for 10 min, followed by cooling on ice for 5 min. After that, the tubes were centrifuged at 20,000* g* for 15 min. Supernatants were collected, and 100 μL was transferred to 96-well plates, along with 40 μL of 10% ammonium hydroxide to neutralize the acid. The absorbance was measured using a spectrophotometer (Epoch; Bio-Tek, Winooski, VT) at a wavelength of 405 nm. The formation of the mineralized matrix was expressed as absorbance and was calibrated by those obtained from non-exposed cultures. The experiments were conducted independently two times with triplicate samples each time.

### ENPP1 Activity

ENPP1 activity was measured colorimetrically using the chromogenic substrate p-nitrophenyl thymidine 5′-monophosphate, as previously described by Ferreira et al. [35]. After washing with PBS, cells were lysed in a buffer containing 1% Triton X-100 and 200 mM Tris–HCl (pH 8.0). 50 μL of 1 mg/mL thymidine 5′-monophosphate p-nitrophenyl ester (Sigma-Aldrich), diluted in Milli-Q water, was added to 50 μL of cell lysate and incubated for 1 h at 37 °C. The reaction was stopped by adding 200 μL of 0.1M NaOH, and absorbance was measured at 405 nm using a spectrophotometer.

### Quantitative RT-PCR

Samples used for quantitative PCR were collected at days 1, 3, 5, and 7, and the RNA were extracted according to manufacturer’s directions (RNAspin Mini Kit, GE Healthcare) and stored at −70 °C. The RNA concentration and purity were assessed spectrophotometrically (260/280 ratio) (Nanodrop 1000, Wilmington, NC, USA). Reverse transcription of total RNA was DNase treated (Turbo DNA-frees; Ambion Inc., Austin, TX, USA) and 1 μg was used for cDNA synthesis (Roche Diagnostic, Indianapolis, USA). Quantitative real-time PCR was performed with FastStart DNA Master PLUS SYBR Green (Roche Diagnostics), using a LightCycler System (Roche Diagnostics). The reaction product was quantified with the Relative Quantification tool, using GAPDH as the reference gene. Negative controls with SYBR Green PCR Master Mix and water were performed for all reactions. Targets analyzed were *Osx* (Sp7), *Runx2*, *Col1a1*, *Bsp* (IBSP), *Bglp* (osteocalcin), and ENPP1. All gene sequences are shown in Table 1.

**Table 1:** Primer gene sequencing

Target gene	Foward sequence	Reverse sequence
OSX	GCGTCCTCCCTGCTTGAG	TGCCCCCATATCCACCACTA
RUNX2	GAGGCGGTCAGAGAACAAAC	GGAAGTGGACGAGGCAAGAG
COL1A1	CCAGAAGAACTGGTACATCAGCAA	GGACATCAGGCGCAGGAA
BSP	ACGATTTCCAGTTCAGGGCAG	GCCCAGTGTTGTAGCAGAAAG
BGLAP	ACCTGTATCAATGGCTGGGAG	AGCAGAGCGACACCCTAGAC
ENPP1	TTGCAAAGGTCGCTGTTTCG	GCCAGACTTGAGGCCTTCAT
GAPDH	ACCCACTCCTCCACCTTTGA	TGTTGCTGTAGCCAAATTCGTT

### Immunofluorescence

Cells were fixed at 1, 5, 7, and 10 days after induction of osteogenic stimulation, or not (OM−), using cold methanol (Merck, Darmstadt, Germany) for 5 min at −20 °C, followed by rinsing in 0.1 M sodium phosphate buffer (PB, pH 7.2). Non-specific binding was minimized by incubating the samples with 5% skimmed milk in PB for 30 min. Primary antibody incubation was performed using anti-ENPP1 (1:100, bs-1760R, Bioss). After washing in PB (3 × 5 min), cells were incubated with an Alexa Fluor 594-conjugated goat anti-rabbit secondary antibody (1:200, Molecular Probes, Invitrogen/Life Technologies, Carlsbad, CA, USA) for 60 min at room temperature in a humidified chamber. Slides were mounted with Vectashield (Vector Laboratories, Burlingame, CA, USA) and visualized under a Zeiss Axioskop 2 epifluorescence microscope (Carl Zeiss, Göttingen, Germany) equipped with an Axiocam MRm digital camera.

ENPP1 immunofluorescence was quantified by counting the cells in four microscopic fields per biological replicate. In each field, 25 DAPI-positive cells were selected and evaluated for ENPP1 immunoreactivity, resulting in a total of 100 cells analyzed per replicate. The proportion of ENPP1-positive cells was calculated relative to the number of DAPI-positive cells evaluated in each field and expressed as the percentage of ENPP1-positive cells. Three independent biological replicates were analyzed (n = 3).

### Statistical Analysis

Normality was assessed by the Shapiro–Wilk test. Two-group comparisons used Student’s t-test or Mann–Whitney; multi-group comparisons used one-way ANOVA with Tukey’s post-hoc test, or Kruskal–Wallis with Dunn’s post-hoc, as appropriate. Two-factor experimental designs were analyzed by two-way ANOVA with Tukey’s correction. For IF quantification, data were analyzed by two-way ANOVA considering treatment and time as experimental factors, followed by Šídák’s multiple-comparisons test. Significance was set at *p* < 0.05. Data are presented as mean ± SD. Analyses were performed in GraphPad Prism 11.

## Results

### TPPU Accelerates Early Bone Regeneration in Critical-Size Calvarial Defects

**Fig. 1:**
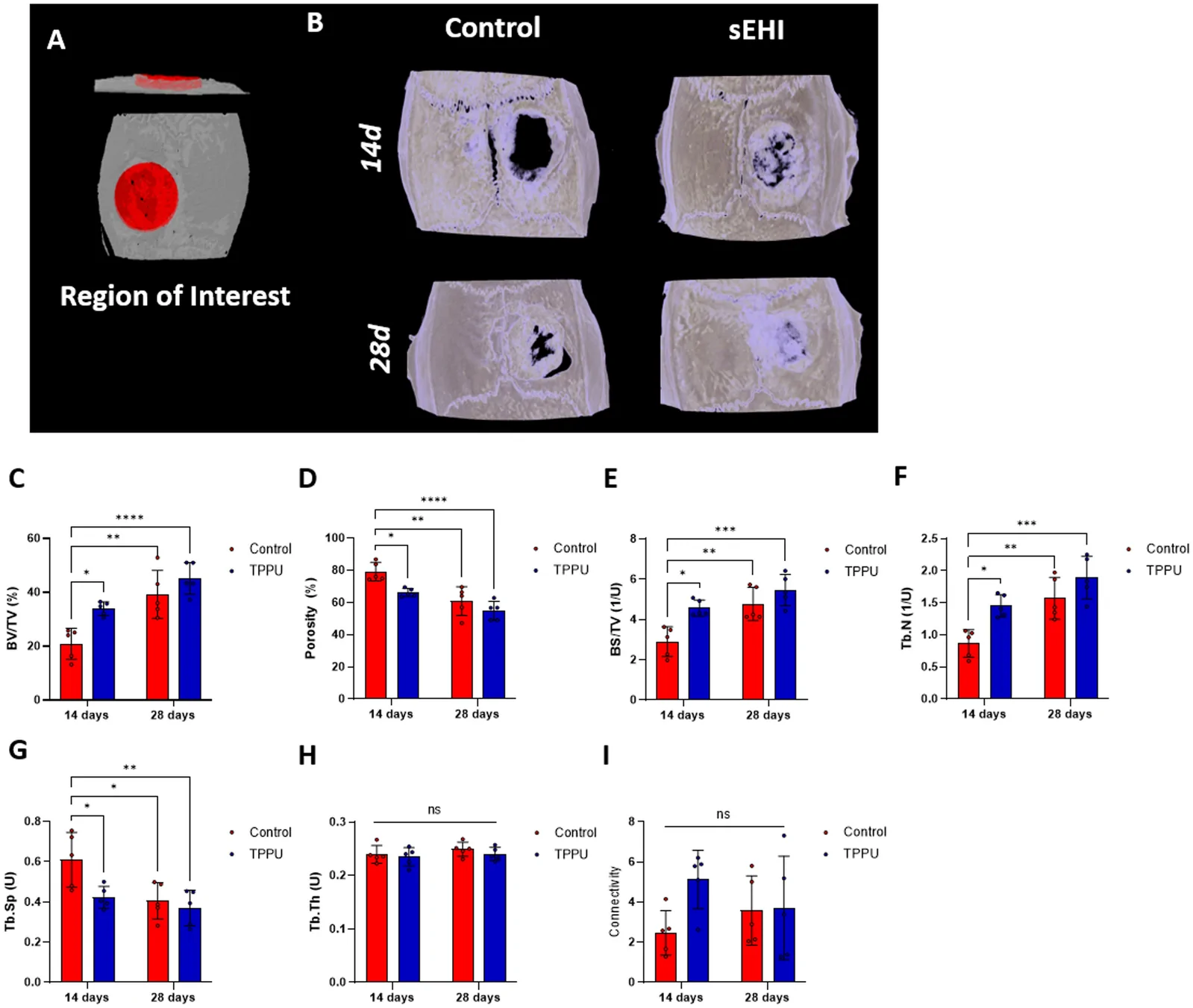
TPPU accelerates early bone regeneration in critical-size calvarial defects. **A** volume of interest (VOI) covering the entire 5-mm bicortical defect; bone within the VOI was segmented and three-dimensional morphometric parameters calculated in CTAnalyzer (Bruker, Belgium). **B** representative micro-CT 3D reconstructions at 14 and 28 days. The TPPU group exhibits centripetal defect filling at 14 days, whereas controls show predominantly peripheral ossification. **C** BV/TV (%); **D** total porosity (Po[tot], %); **E** bone surface density (BS/TV, %); **F** trabecular number (Tb.N, 1/mm); **G** trabecular separation (Tb.Sp, mm); **H** trabecular thickness (Tb.Th, mm); **I** connectivity density (Conn.Dn, 1/mm^3^). Red bars indicate non-treated animals. Blue bars indicate animals treated with TPPU. Data are mean ± SD; n = 6 per group. *p < 0.05; **p < 0.01; ***p < 0.001; ****p < 0.0001

**Fig. 2:**
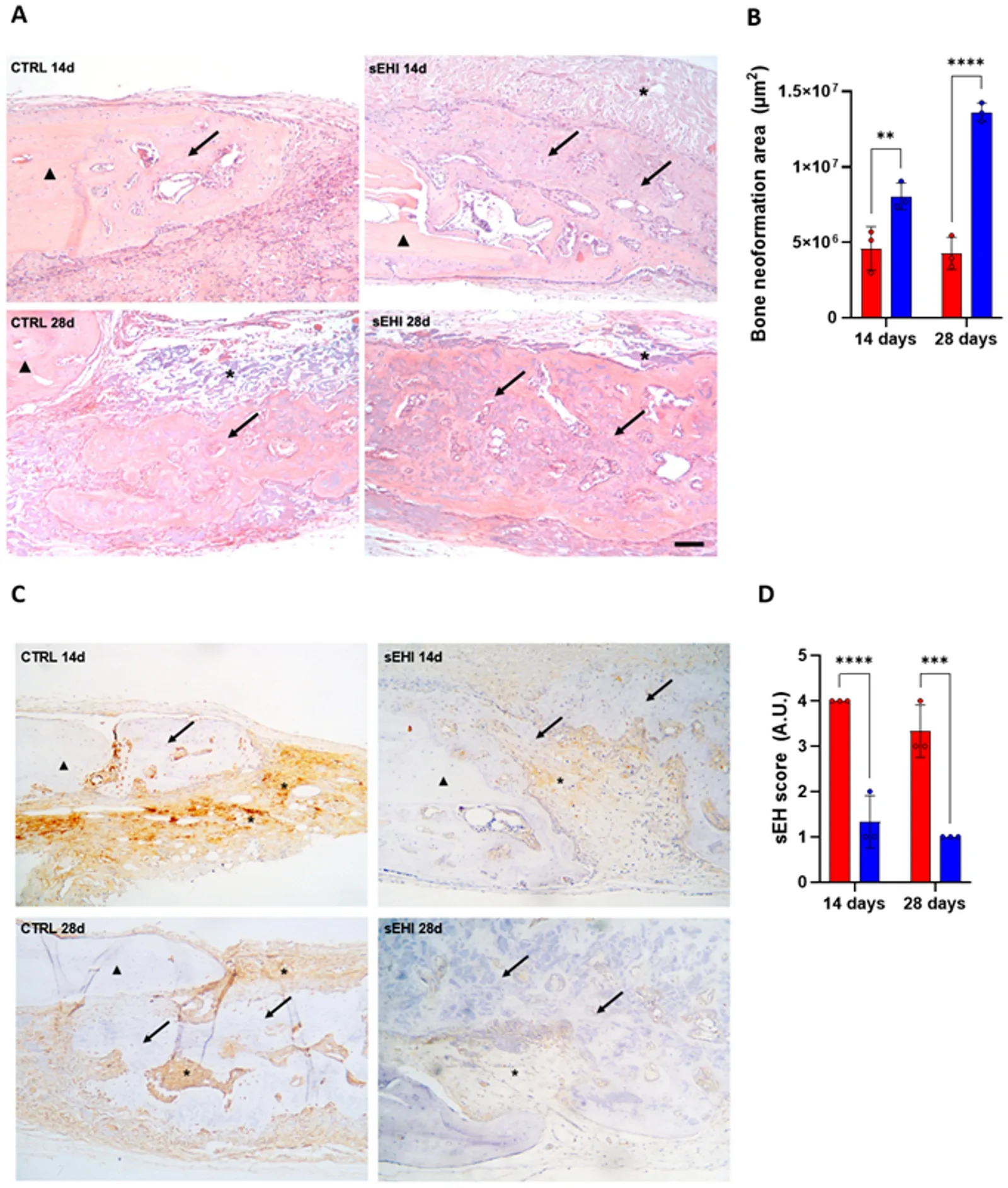
Histomorphometric and immunohistochemical characterization of TPPU-treated defects. **A** representative H&E sections of critical-size defects in control and TPPU-treated (sEHi) groups. Arrows: newly formed bone within the implantation bed; arrowheads: lamellar bone; asterisks: residual collagen membrane. Scale bar = 400 μm. **B** quantitative bone neoformation area (μm^2^). **C** representative sEH immunostaining; TPPU-treated tissue shows reduced sEH labelling at both time points. **D** quantitative immunostaining score for sEH (A.U.). Red bars indicate non-treated animals. Blue bars indicate animals treated with TPPU. Data are mean ± SD. **p < 0.01; ***p < 0.001; ****p < 0.0001

To determine whether systemic sEH inhibition enhances bone formation in critical-size defects, Wistar rats with bilateral 5-mm bicortical calvarial defects received TPPU (10 mg/kg/day, p.o.) or vehicle for 14 or 28 days (Fig. 1A). Three-dimensional micro-CT reconstructions showed that, at 14 days, the TPPU group exhibited substantial centripetal defect filling, whereas controls displayed only peripheral ossification (Fig. 1B). Quantitative morphometry confirmed a significant increase in BV/TV, BS/TV and Tb.N and a significant reduction in total porosity (Po[tot]) and trabecular separation (Tb.Sp) at 14 days (all *p* < 0.05; Fig. 1C–G). Trabecular connectivity (Conn.Dn) showed an increasing trend without reaching significance (*p* > 0.05; Fig. 1I). Histomorphometric measurement of newly formed bone area corroborated these findings: the TPPU group displayed significantly greater bone neoformation than controls at both 14 and 28 days (*p* < 0.05; Fig. 2A and B).

By 28 days, however, the structural advantage in the treated group was no longer detectable: micro-CT parameters in the control group caught up significantly between 14 and 28 days (*p* < 0.01), whereas the TPPU group showed no further significant gains over the same interval (*p* > 0.05). Together with the histological progression, these data define a discrete early-phase window during which TPPU accelerates ossification of the defect.

### TPPU Sustains Suppression of sEH Expression in Regenerating Bone

Immunohistochemical labelling of sEH in regenerating bone (Fig. 2C and D) showed markedly elevated sEH expression in untreated animals at 14 days, consistent with persistent enzymatic activity during the inflammatory phase of bone healing (*p* < 0.0001 vs TPPU). At 28 days sEH expression naturally declined in controls but remained significantly higher than in TPPU-treated animals (*p* < 0.001), indicating sustained pharmacodynamic engagement of the target over the entire experimental window.

### Spatial Transcriptomics Reveal Coordinated Osteogenic Activation and ENPP1 Upregulation in TPPU-Treated Regenerating Tissue

**Fig. 3:**
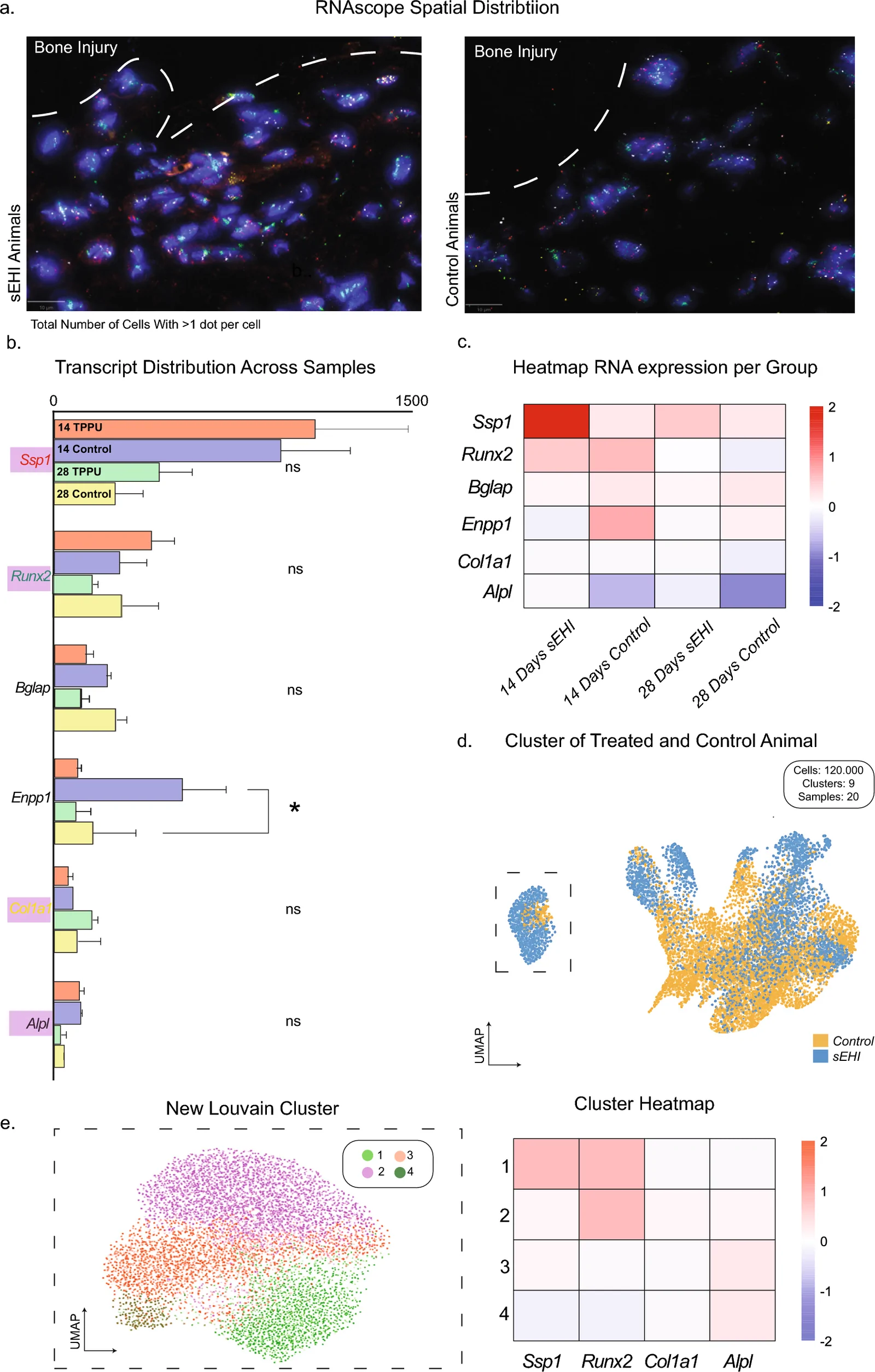
Spatial transcriptomics identifies a TPPU-specific osteogenic program with ENPP1 upregulation. **A** representative multiplex RNAscope images (40 ×) of the bone defect region (dashed line) showing *Spp1* (red), *Runx2* (green), *Col1a1* (yellow) and *Alpl* (blue) at single-transcript resolution. **B** per-cell transcript quantification after subcellular segmentation. ENPP1 expression was significantly higher in the TPPU-treated group than in controls (*p* = 0.03 at 14 days). **C** heatmap of bulk-level relative expression of selected osteogenic and inflammatory markers across groups and time points. **D** UMAP projection of 120,000 cells from 20 samples (9 unsupervised clusters); inset highlights the cluster overwhelmingly composed of TPPU-derived cells. **E** louvain subclustering of the TPPU-enriched population reveals four transcriptionally distinct subpopulations, including one co-expressing *Spp1* and *Runx2* (active osteoprogenitors) and another with high *Runx2* alone, consistent with intermediate osteoblast-like states. Red bars indicate non-treated animals. Blue bars indicate animals treated with TPPU

To resolve the cellular and transcriptional landscape underlying TPPU-driven osteogenesis, we performed multiplex RNAscope on regenerated calvarial sections, profiling *Spp1, Runx2, Col1a1, Alpl, Bglap* and ENPP1 at single-cell resolution (Fig. 3A). TPPU-treated samples displayed a markedly more focal, intracellular distribution of *Spp1* and *Runx2* transcripts within the defect margin, while controls showed a diffuse, lower-intensity pattern dispersed across the surrounding tissue. At the per-cell level, ENPP1-positive cells were significantly more abundant in the TPPU group at 14 days than in controls at either time point (*p* = 0.03; Fig. 3B), whereas *Spp1, Runx2, Bglap, Col1a1* and *Alpl* per-cell counts trended upward without reaching individual significance—indicating that TPPU selectively expanded the ENPP1-expressing cell pool at the early phase of repair.

When the analysis shifted from per-cell intensity to the proportion of cells expressing each marker, a coordinated osteogenic signature emerged: TPPU-treated animals at 14 days showed an increased number of *Spp1*⁺, *Runx2*⁺ and ENPP1⁺ cells, while *Col1a1* and *Alpl* remained low across all groups, with the lowest values in 28-day controls (Fig. 3C). Unsupervised UMAP projection of 120,000 cells from 20 samples partitioned the dataset into nine clusters and identified one cluster overwhelmingly composed of TPPU-derived cells (Fig. 3D), suggesting treatment-specific transcriptional reprogramming. Louvain subclustering of this population (Fig. 3E) resolved four transcriptionally distinct subpopulations. Two of these displayed osteoprogenitor-like profiles, one co-expressing *Spp1* and *Runx2* and another marked by high *Runx2* alone, consistent with active early-to-mid osteoblastic differentiation states, whereas the remaining two subpopulations showed low expression of the osteogenic panel [27]. Together, these data identify ENPP1 as a candidate effector positioned within an early, TPPU-induced osteogenic program.

### TPPU Upregulates ENPP1 Transcript and Protein in Human Osteoblasts

**Fig. 4:**
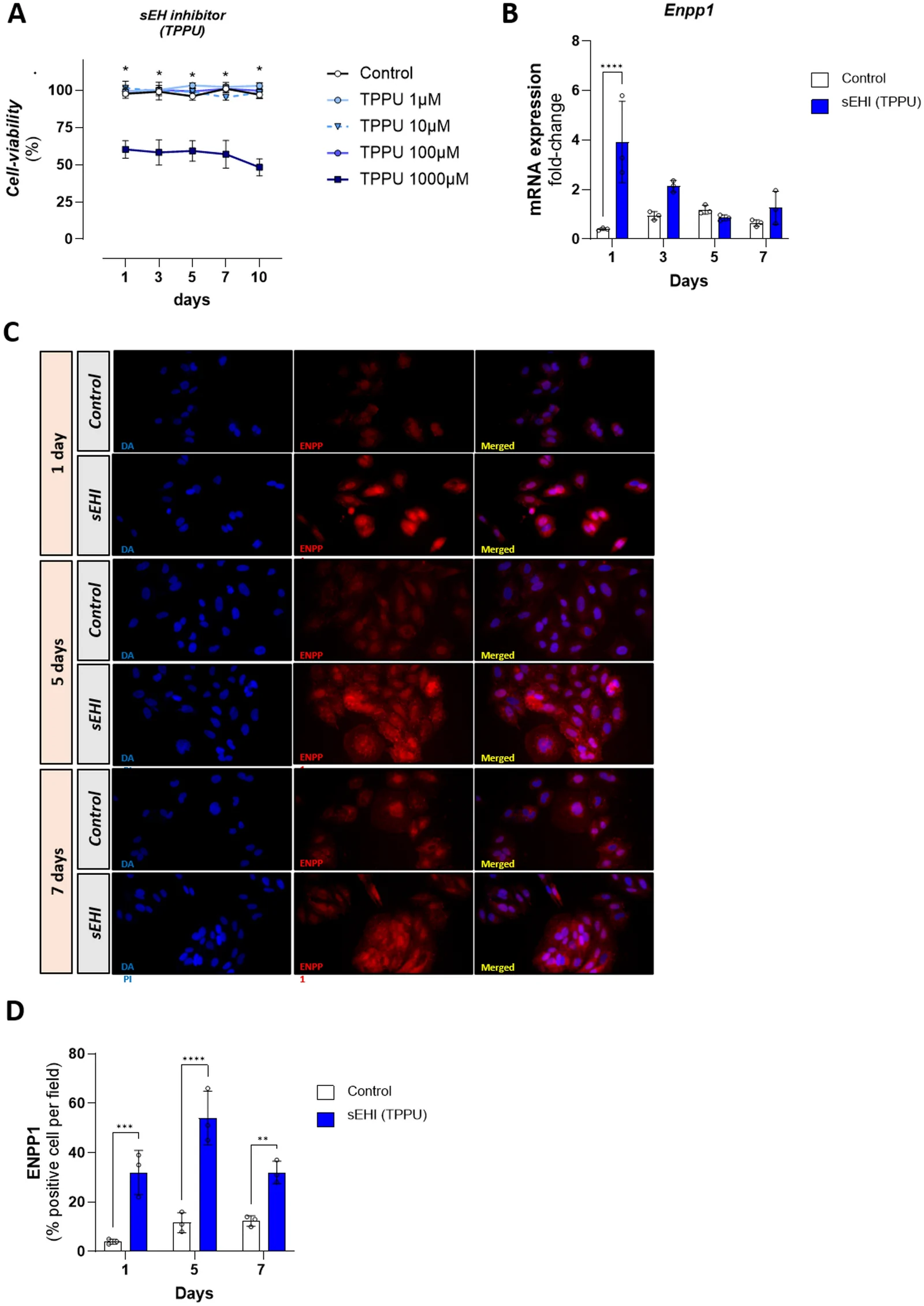
TPPU upregulates ENPP1 transcript and protein in human osteoblasts. **A** MTT viability of SAOS-2 cells exposed to TPPU (1–1000 μM) at 1, 3, 5, 7 and 10 days; only 1000 μM was cytotoxic. **B** relative ENPP1 mRNA expression following TPPU at 100 μM at 1, 3 and 7 days. ENPP1 was significantly upregulated at 24 h, with sustained but non-significant elevation at later time points. **C** representative immunofluorescence images showing increased ENPP1 protein in TPPU-treated cultures at 1, 5 and 7 days. **D** ENPP1-positive cells were quantified by immunofluorescence at days 1, 5, and 7. Four microscopic fields were analyzed per biological replicate. Data are expressed as the percentage of ENPP1-positive cells and presented as mean ± SD of three independent biological replicates (n = 3). Statistical analysis was performed using two-way ANOVA (treatment × time), followed by Šídák’s multiple-comparisons test for comparisons between treatments within each time point. Data are mean ± SD. *p < 0.05; **p < 0.01; ***p < 0.001; ****p < 0.0001

To dissect the cellular mechanism, we turned to human SAOS-2 osteoblastic cultures. MTT analysis across 1, 3, 5, 7 and 10 days showed that TPPU at 1–100 µM did not affect mitochondrial activity, whereas the highest dose tested (1000 µM) produced cytotoxicity (Fig. 4A). Furthermore, pharmacological sEH inhibition (TPPU) at 100 µM, significantly upregulated ENPP1 mRNA (*p* < 0.001; Fig. 4B) in 24 h. Besides, ENPP1 transcripts remained elevated at 3 and 7 days without reaching statistical significance. Lastly, immunofluorescence confirmed increased ENPP1 protein in TPPU-treated cultures at all three time points (Fig. 4C), consistent with the in vivo single-cell transcriptomic finding. As shown in Fig. 4D, immunofluorescence quantification demonstrated that sEH inhibition significantly increased ENPP1 expression at all three time points analyzed (*p* < 0.001).

### Pharmacological Inhibition of ENPP1 Abolishes Osteogenic Differentiation and Matrix Mineralization

**Fig. 5:**
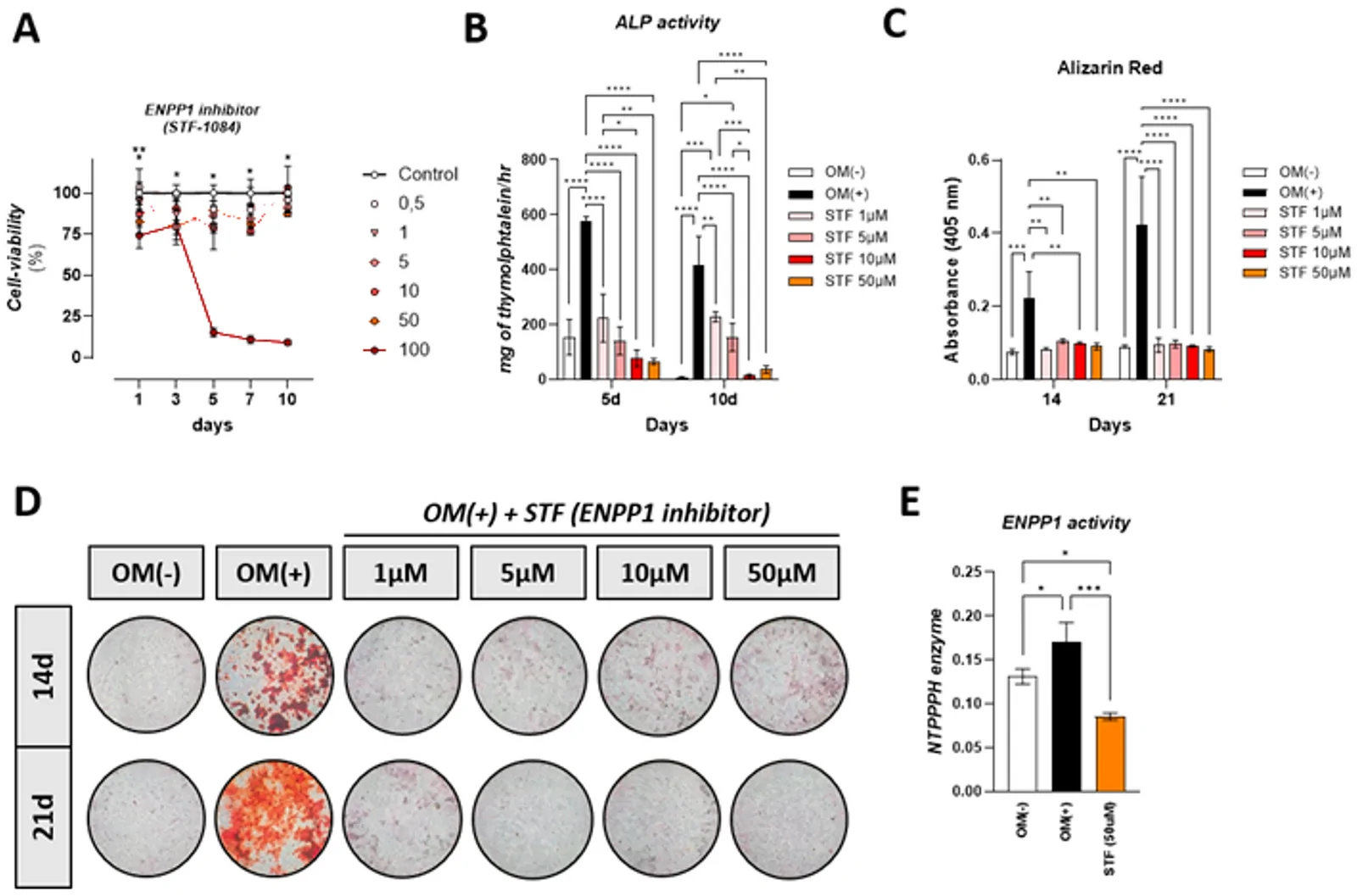
Pharmacological ENPP1 inhibition abolishes osteogenic differentiation and mineralization. **A** MTT viability of SAOS-2 cells exposed to STF-1084 (0.5–100 μM); only 100 μM was cytotoxic. **B** ALP activity under non-osteogenic [OM( −)] or osteogenic [OM( +)] conditions, with STF-1084 (1–50 μM) at 5 and 10 days. ENPP1 inhibition significantly decreased ALP activity in OM( +) cultures. **C** alizarin red quantification at 14 and 21 days showing reduced calcium deposition in STF-1084-treated osteogenic cultures. **D** representative Alizarin Red images. **E** ENPP1 enzymatic activity in OM( −), OM( +), and OM( +) + STF-1084 cultures, confirming on-target engagement of STF-1084. Data are mean ± SD. *p < 0.05; **p < 0.01; ***p < 0.001; ****p < 0.0001

We next tested whether ENPP1 activity itself is required for osteogenic differentiation in this system using the selective inhibitor STF-1084. STF-1084 at 0.5–50 µM did not affect SAOS-2 viability over 10 days, while 100 µM was cytotoxic (Fig. 5A). Under osteogenic medium [OM( +)], STF-1084 dose-dependently and significantly reduced ALP activity at both 5 and 10 days compared with OM( +) controls (*p* < 0.05; Fig. 5B). Mineralized matrix deposition, assessed by Alizarin Red staining at 14 and 21 days, was likewise significantly reduced across all tested STF-1084 concentrations (*p* < 0.05; Fig. 5C and D). To confirm on-target engagement, ENPP1 enzymatic activity was quantified: osteogenic stimulation increased ENPP1 activity, and STF-1084 significantly suppressed this activity (*p* < 0.05; Fig. 5E), confirming that the differentiation defect is attributable to loss of ENPP1 catalytic function. Together, these results identify ENPP1 as functionally required for osteoblast maturation and matrix mineralization in this human osteoblastic system, in agreement with prior genetic evidence [29].

### ENPP1 Activity is Required for the Osteogenic Effect of TPPU

**Fig. 6:**
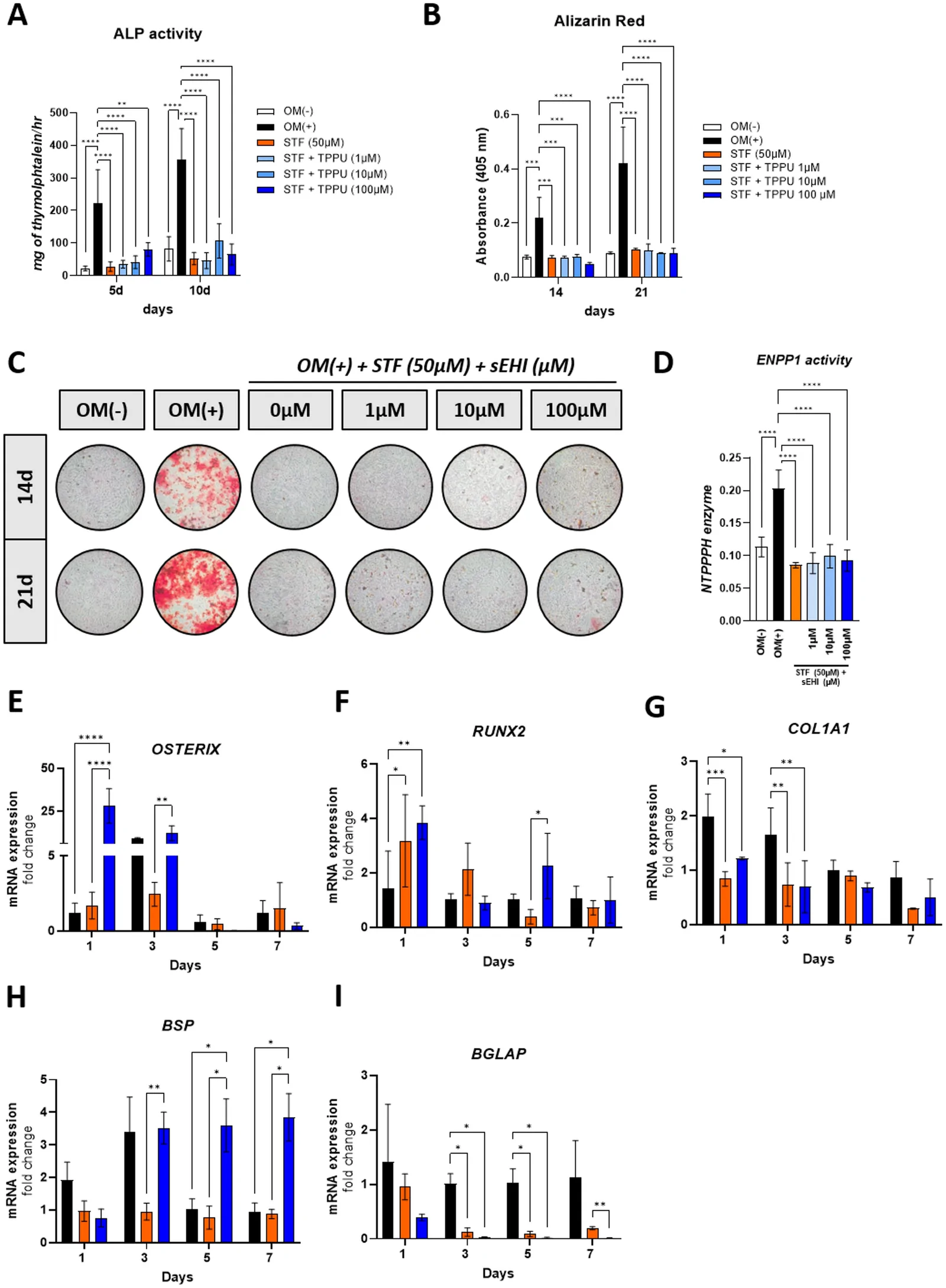
ENPP1 activity is required for the osteogenic effect of TPPU. **A** ALP activity at 5 and 10 days in OM( −), OM( +) and OM( +) + STF-1084 + TPPU cultures. The combination significantly reduced ALP activity vs OM( +) at both time points. **B** alizarin red quantification at 14 and 21 days showing reduced mineralized matrix in the combination group. **C** representative alizarin red images. **D** ENPP1 enzymatic activity, confirming that TPPU does not reverse STF-1084-mediated ENPP1 inhibition. **E**–**H** relative mRNA expression of *Osx*, *Runx2*, *Col1a1* and *Bsp*. The combination group shows preserved or amplified expression of *Osx*, *Runx2* and *Bsp*, with reduced *Col1a1* at early time points—a dissociation between osteogenic transcription factors and functional mineralization that places ENPP1 downstream of the transcriptional program. Data are mean ± SD. *p < 0.05; **p < 0.01; ***p < 0.001; ****p < 0.0001

To formally test whether the pro-osteogenic action of sEH inhibition depends on ENPP1, we co-treated osteogenic SAOS-2 cultures with STF-1084 and TPPU. The combination significantly reduced ALP activity at 5 and 10 days compared with OM( +) (*p* < 0.05; Fig. 6A) and significantly reduced Alizarin Red-positive mineralization at 14 and 21 days (*p* < 0.05; Fig. 6B and C). Crucially, ENPP1 enzymatic activity remained suppressed in the combination group (*p* < 0.05 vs OM( +); Fig. 6D), demonstrating that TPPU could not rescue ENPP1 activity once it was pharmacologically blocked, and consistent with TPPU acting upstream of ENPP1.

Despite the loss of mineralization, the OM( +) + STF + TPPU group displayed a distinctive transcriptional signature: OSX expression was significantly upregulated at 1 and 3 days versus all other groups (*p* < 0.05; Fig. 6E), *Runx2* expression was higher than in STF-treated cultures at 5 days (Fig. 6F;* p* < 0.05), and *Bsp* expression was significantly elevated at 3, 5 and 7 days versus STF alone (Fig. 6H; *p* < 0.05). *Cola1a1* was significantly reduced in both STF-treated and combination groups at 1 and 3 days versus OM( +) (Fig. 6G; *p* < 0.05). The dissociation between preserved transcription-factor expression and abolished mineralization is consistent with ENPP1 acting downstream of the osteogenic transcriptional program; the underlying mechanism was not determined here.

**Fig. 7:**
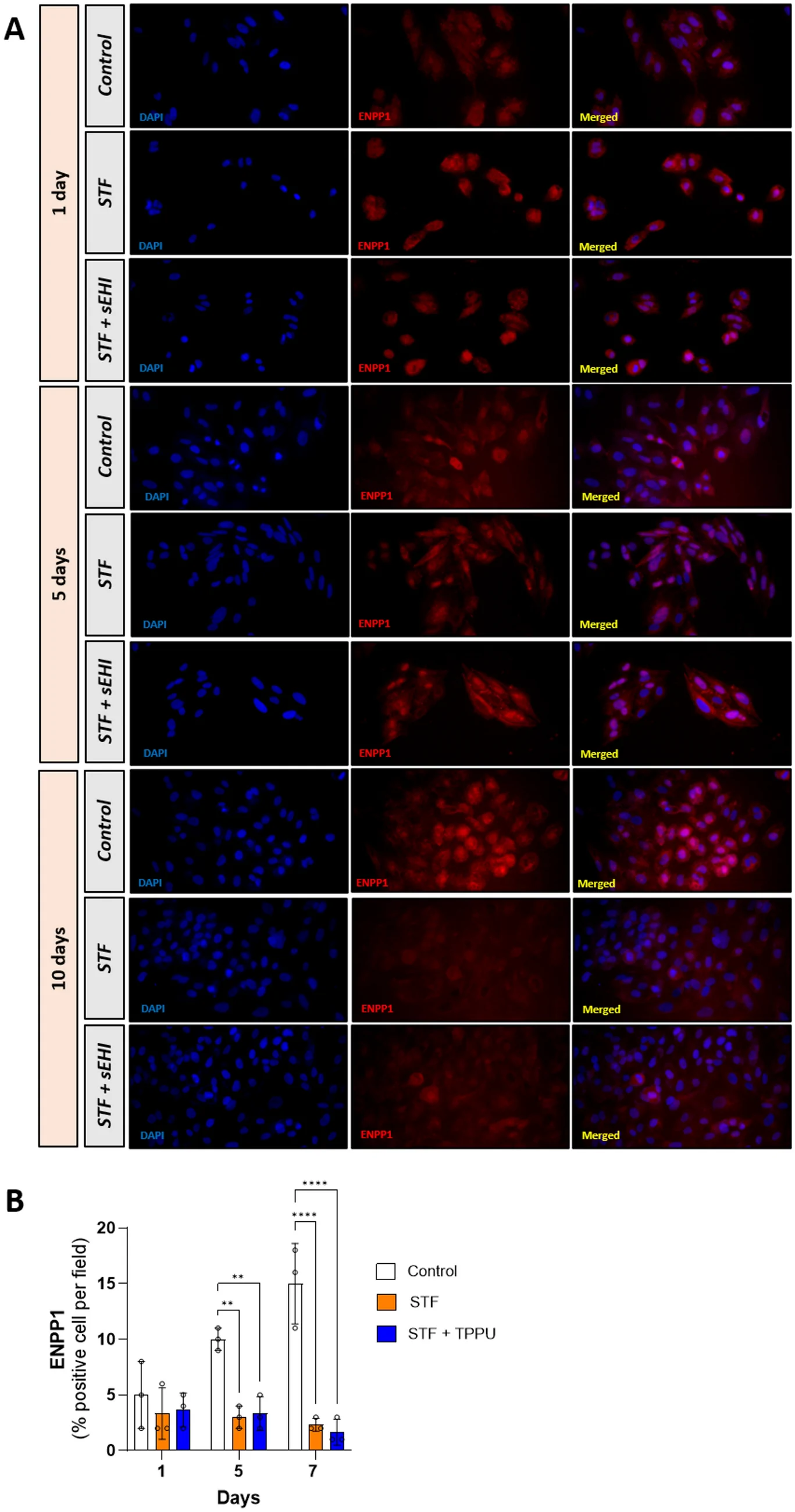
sEH inhibition fails to restore ENPP1-immunopositive cells under ENPP1 blockade. **A** SAOS-2 cells were cultured with or without osteogenic induction (OM− and OM+, respectively) and treated with STF (OM+ and 50 μM of STF-1084), or STF+sEHI (OM+ and 100 μM of TPPU) for 1, 5 and 10 days. DAPI was used to nuclear stain (blue). Merged images were shown in the last column. Representative immunofluorescence images in each group were provided. Magnification: ×20. Scale bar: 100 μm. **B** ENPP1-positive cells were quantified by immunofluorescence at days 1, 5, and 10. Four microscopic fields were analyzed per biological replicate. Data are expressed as the percentage of ENPP1-positive cells and presented as mean ± SD of three independent biological replicates (n = 3). Statistical analysis was performed using two-way ANOVA (treatment × time), followed by Šídák’s multiple-comparisons test for comparisons between treatments within each time point. **p < 0.01; ****p < 0.0001

Last, immunofluorescence staining was performed to assess ENPP1-immunopositive cells (Fig. 7A). Under non-osteogenic conditions, ENPP1 immunoreactivity was low, consistent with the reduced osteogenic commitment of control cultures. As expected, osteogenic induction increased the number of ENPP1-immunopositive cells, supporting the association between ENPP1 expression and the osteogenic/mineralizing phenotype. However, pharmacological inhibition of ENPP1 with STF-1084 attenuated this osteogenic-induced increase in ENPP1-positive cells. Figure 7B demonstrated ENPP1 positive cells increased progressively in the control group, reaching significantly higher levels at days 5 and 7 compared with both STF and STF + TPPU groups. At day 5, ENPP1 expression was significantly higher in controls than in the STF and STF + TPPU groups (*p* < 0.01), with an even greater difference observed at day 7 (*p* < 0.001). No significant differences were observed among groups at day 1.

Previous findings demonstrated that TPPU enhances ENPP1 expression both in vitro and in vivo. In the present experimental setting, however, TPPU failed to restore ENPP1 immunoreactivity when ENPP1 activity was pharmacologically inhibited by STF-1084. This observation suggests that the osteogenic effects promoted by TPPU may require functional ENPP1 signaling. Importantly, because STF-1084 primarily inhibits ENPP1 enzymatic activity rather than necessarily suppressing ENPP1 gene or protein expression, these findings should be interpreted as evidence that ENPP1 activity is required for the full TPPU-induced osteogenic response, rather than as definitive proof that STF-1084 directly blocks ENPP1 expression. Although STF-1084 is a selective ENPP1 inhibitor and on-target engagement was confirmed functionally in SAOS-2 cells, its selectivity against other ENPP family members and ectonucleotidases was not profiled in this cell line, and the colorimetric NTPPPH assay used is not exclusive to ENPP1. Contributions from related ecto-enzymes to the phenotype observed therefore cannot be formally excluded.

Collectively, in vivo and in vitro datasets converge on a coherent model in which sEH inhibition by TPPU enhances early bone regeneration through ENPP1-dependent osteoblast maturation. ENPP1 is required at a step downstream of osteogenic transcription factors and upstream of matrix mineralization, and pharmacological loss of ENPP1 cannot be compensated for by sEH inhibition.

## Discussion

The key finding of this study is that systemic pharmacological inhibition of soluble epoxide hydrolase enhances early bone regeneration in critical-size calvarial defects through an ENPP1-dependent osteogenic program. Three complementary observations are consistent with this model, which remains to be confirmed genetically: (i) TPPU accelerates ossification in the first 14 days, with a quantitative early-phase advantage in BV/TV, BS/TV, Tb.N, Po[tot] and Tb.Sp that is no longer detectable by 28 days; (ii) single-cell-resolved spatial transcriptomics identifies an early, coordinated upregulation of *Spp1, Runx2* and ENPP1, with TPPU-driven enrichment of an osteoprogenitor-like cluster co-expressing *Spp1, Runx2*, *Col1a1* and *Alpl*; and (iii) in human osteoblasts, TPPU upregulates ENPP1, pharmacological inhibition of ENPP1 abolishes mineralization, and combined sEH/ENPP1 blockade fails to rescue mineralization despite preserved or amplified osteogenic transcription factor expression. These data identify ENPP1 as a candidate downstream effector of the sEH/EpFA axis, and define a discrete early-phase window for further investigation.

The early-phase nature of the in vivo effect mirrors the known transient biology of EETs and other epoxy-fatty acids, which act in a pulsatile manner to drive inflammation resolution and early tissue regeneration [11, 13, 36, 37]. In our model, sustained reduction of sEH immunolabelling at 14 and 28 days (Fig. 2C and D) confirms continuous pharmacodynamic engagement of the target, yet the regenerative advantage is concentrated in the first phase of healing, consistent with a window during which a higher EpFA tone is required to bias the local microenvironment toward osteogenic commitment [7, 18]. Beyond 14 days, untreated animals appear to “catch up” through normal endogenous remodeling, narrowing the structural gap. Such an early-window pharmacology is therapeutically attractive since it suggests that brief perioperative sEH inhibition may suffice to accelerate consolidation without requiring chronic dosing.

The most novel mechanistic contribution of this work is the identification of ENPP1 as a downstream effector of the sEH/EpFA axis. ENPP1 has long been recognized as a regulator of pyrophosphate metabolism and tissue mineralization [28]; however, ENPP1 also has a context-dependent osteogenic role and is required for osteoblast differentiation in vitro [29]. Our data extend this dual-role concept to a regenerative in vivo setting and a pharmacological framework, TPPU upregulates ENPP1 in regenerating bone and in osteoblastic cultures; selective ENPP1 inhibition collapses ALP activity and matrix mineralization; and the combination of sEH inhibitor and ENPP1 inhibition cannot rescue matrix formation, even though osteogenic transcription factors are preserved or upregulated. This dissociation between transcription-factor expression (*Osx, Runx2, Bsp*) and matrix mineralization positions ENPP1 at a post-transcriptional, maturation-gating step in the osteogenic cascade a step that the sEH/EpFA axis modulates in an ENPP1-dependent fashion.

These findings integrate naturally with the broader EpFA-bone literature. EETs engage ROBO1/SLIT3 signaling to couple osteogenesis with angiogenesis [25], regulate macrophage polarization through PPAR-γ [26], promote pro-resolving lipid mediators and Treg/M2 phenotypes [7, 16, 17], and stabilize endogenous epoxy-fatty acids that support osteoblast metabolism [22]. Recent evidence further demonstrates that sEH inhibition reverses periodontitis-associated bone loss by upregulating EMCN and inhibiting osteoclasts [27] and preserves alveolar bone in estrogen-deficient periodontitis [24], while restraining osteoclast differentiation [23]. The present study complements these reports by adding a pro-anabolic axis: in critical-size defects, sEH inhibition is accompanied by increased ENPP1 expression in regenerating bone and in osteoblastic cultures, and ENPP1 activity is necessary for mineralisation in vitro.

Translationally, several orally active sEH inhibitors have advanced through clinical pharmacology with favorable safety profiles, including EC5026 in healthy volunteers [20] and randomized trials in aneurysmal subarachnoid hemorrhage [19, 21]. The combination of a transient, early-window mechanism, a clean cellular safety profile and an identified molecular effector (ENPP1) supports the development of sEH inhibitors as bone-anabolic adjuncts in craniofacial reconstruction, where the standard of care still relies on autografts, xenografts or alloplastic substitutes with limited regenerative capacity [1–5]. Pairing sEH inhibition with biomaterial scaffolds, controlled-release platforms or pro-resolving lipid mediator strategies [8–10, 38, 39] is a logical next step.

The relationship between ENPP1 and mineralisation is, however, context-dependent and not unidirectional. By hydrolysing extracellular ATP, ENPP1 generates PPi, a potent inhibitor of hydroxyapatite formation [28, 40], and inactivating ENPP1 mutations cause generalised arterial calcification of infancy, a disorder of ectopic mineralisation [41]. Mineral deposition is therefore governed by the balance between PPi generation by ENPP1 and its hydrolysis by TNAP and PHOSPHO1, rather than by the level of any single enzyme [42, 43], and increased ENPP1 expression does not by itself indicate a pro-mineralising effect. Against this background, the informative observation in the present study is not the increase in ENPP1 expression but the consequence of blocking its activity: in SAOS-2 cells, ENPP1 inhibition reduced ALP activity and abolished mineralised matrix deposition, indicating that in this osteoblastic context ENPP1 acts predominantly at the level of osteoblast differentiation and maturation, in agreement with previous genetic evidence [29]. Whether the same directionality applies to other cell types, differentiation stages or pathological settings remains to be determined.

Some limitations should be acknowledged. First, the in vitro model relies on the SAOS-2 line. Although SAOS-2 cells are a widely used human osteoblast-like model that expresses ALP, osteogenic transcription factors and a robust mineralising phenotype, they are derived from an osteosarcoma and carry a transformed genotype; their baseline osteogenic commitment, differentiation kinetics and signalling responses do not fully reproduce those of primary human osteoblasts or of MSC-derived osteoblasts. Second, although sEH inhibition is known to promote inflammation resolution, no inflammatory endpoint (cytokine profiling, macrophage polarisation or lipid mediator quantification) was measured here. Our data therefore support an osteogenic and mineralisation-related mechanism, but do not establish whether, or to what extent, modulation of the local inflammatory response contributes to the accelerated repair observed. Third, ENPP1 was interrogated as a pre-specified candidate within a targeted six-gene spatial panel rather than identified by an unbiased screen; other effectors of the sEH/EpFA axis in bone that were not represented in the panel cannot be excluded, and genome-wide profiling of TPPU-treated regenerating tissue is warranted. Although TPPU is a highly selective, low-nanomolar inhibitor of sEH, the micromolar concentrations required in cell culture, which reflect serum binding and free-drug availability rather than enzyme affinity, mean that off-target activity in SAOS-2 cells cannot be formally excluded. Cell-line-specific selectivity profiling was not performed, and the viability screen presented here excludes gross cytotoxicity but not subtler off-target effects. Also, EET-antagonist experiments were not performed. Consequently, although sEH inhibition stabilises EETs and other epoxy-fatty acids, we cannot attribute the effects reported here specifically to EET signalling as opposed to other consequences of sEH inhibition, such as the accumulation of additional epoxy-fatty acid species, the reduction of downstream diols, or the activity of the N-terminal phosphatase domain of the enzyme. Finally, our in vitro rescue experiment establishes ENPP1 dependence, in vivo confirmation using pharmacological or genetic ENPP1 inhibition in the calvarial defect model has not yet been performed and represents the natural next step.

In summary, sEH inhibition enhances early calvarial bone regeneration through an ENPP1-dependent osteogenic program, with ENPP1 acting downstream of osteogenic transcription factors to gate matrix mineralization. These findings elevate the sEH/EpFA/ENPP1 axis from a candidate inflammation-resolution pathway to a defined, mechanistically tractable target for bone regenerative pharmacology, and provide a rationale for the clinical translation of sEH inhibitors as bone-anabolic adjuncts in craniofacial reconstruction.

## Data Availability

The data that support the findings of this study are available from the corresponding author upon reasonable request.

## Declarations

### Artificial Intelligence (AI) – Assisted Technology

An AI tool was used solely for English language correction, writing refinement and Graphical abstract generation, without any contribution to the creative or scientific content of the manuscript. After using this tool/service, the author(s) reviewed and edited the content as needed and take(s) full responsibility for the content of the published article.
